# Nano-zeolite-coupled biochar-based phosphorus fertilizer enhances soil phosphorus availability and leaf phosphorus concentration in Moso bamboo forests

**DOI:** 10.3389/fpls.2026.1873995

**Published:** 2026-06-17

**Authors:** Junjie Xie, Jiadong Liu, Zhenhui Jiang, Mouliang Xiao, Jiashu Zhou, Bing Yu, Yongfu Li

**Affiliations:** College of Environmental and Resource Sciences, Zhejiang A&F University, Hangzhou, China

**Keywords:** biochar-based fertilizer, leaf phosphorus concentration, Moso bamboo forest, nano-zeolite, phosphorus availability

## Abstract

**Introduction:**

Phosphorus (P) availability is a major constraint on nutrient management in acidic Moso bamboo (Phyllostachys edulis) forests, where conventional P fertilizers are prone to rapid fixation by soil minerals. Biochar-based P fertilizers can help retain P in soil. Yet it is still unclear whether they can sustain soil P availability and support plant P nutrition over time in acidic forest soils.

**Methods:**

Here, we carried out a 24-month field trial in a subtropical Moso bamboo forest. We compared three P fertilizer treatments: conventional phosphorus fertilizer (CP), biochar-based phosphorus fertilizer (BP), and nano-zeolite-coupled biochar-based phosphorus fertilizer (NBP). Soil P fractions, microbial biomass P, and leaf P concentration were investigated.

**Results and discussion:**

Compared with the CP treatment, the BP and NBP treatments increased soil pH and retained more P in labile and moderately labile pools. The NBP treatment exhibited the strongest positive effects on soil P availability, particularly after 24 months. At this time, NBP reduced the accumulation of HCl-P_i_, a relatively poorly available inorganic P fraction, while maintaining more P in accessible forms, including H_2_O-P_i_, NaHCO_3_-P_i_, NaHCO_3-_P_o_, and NaOH-P_i_. Beyond these physicochemical changes, NBP also enhanced biological P cycling, as indicated by increased microbial biomass P, *phoD* gene abundance, alkaline phosphatase activity, and leaf P concentration. These results suggest that NBP improved P supply through both microbial P retention and organic P mineralization. Overall, the nano-zeolite coupling enhanced the capacity of biochar-based P fertilizer to alleviate soil acidity and sustain P availability through these combined physicochemical and biological processes. The results obtained in this study reveal that NBP is a promising strategy for improving soil P availability and sustaining P nutrition in acidic Moso bamboo forest soils.

## Introduction

Soil phosphorus (P) supply often limits the productivity and ecological function of subtropical Moso bamboo (*Phyllostachys edulis*) forests (Bi et al., 2026). Many Moso bamboo forests occur in the acidic red-soil region of southern China. These soils usually have low pH, abundant Fe and Al oxides, and a strong capacity to fix P (Ma et al., 2025). In acidic soils, fertilizer-derived phosphate can be adsorbed or precipitated by Fe and Al oxides. This process moves P into less available inorganic pools and lowers fertilizer P use efficiency (Chen et al., 2022; Buthelezi-Dube and Sithole, 2025). Acidic soils may also contain high levels of active Al and Mn. These ions can restrict root growth and nutrient uptake, which further worsens P limitation in Moso bamboo forests (Hailai et al., 2024; Ur Rahman et al., 2024). For this reason, nutrient management in these forests needs to keep more P in plant-available forms and reduce its conversion into stable pools.

Biochar-based fertilizers can improve soil conditions while supplying nutrients. Biochar has many pores, a large surface area, active surface groups, and alkaline components (Huang et al., 2023; Premalatha et al., 2023; Adhikari et al., 2024; Sun et al., 2025). In acidic soils, biochar can raise pH, increase cation exchange capacity, and improve water and nutrient retention (Bolan et al., 2023; Liu et al., 2025; Zhou et al., 2026). Biochar can be mixed with inorganic P fertilizer, which slows down the release of phosphorus. This can reduce P leaching and also helps limit the rapid fixation of soluble phosphate in soil (Melo et al., 2022; Wang et al., 2022). Although these effects are beneficial, they may not persist in subtropical red soils because of high rainfall, warm climatic conditions, and strong soil acidity (Liu et al., 2025; Wang et al., 2026). However, the long-term effects of biochar-based P fertilizers on soil P fractions, microbial P mineralization, and plant P uptake in Moso bamboo forests remain poorly understood.

Nano-zeolite can enhance the efficacy of biochar-based fertilizers. Nano-zeolite has a large surface area, microporous structure, and strong ion-exchange capacity. These features help it hold onto mineral nutrients (Legese et al., 2024; Dong et al., 2025). Adding nano-zeolite to biochar-based P fertilizer may increase nutrient adsorption and slow P release. It may also reduce the rapid loss or inactivation of fertilizer-derived P in acidic soils (Khan et al., 2021; Dong et al., 2025). Biochar and nano-zeolite may further improve soil pH buffering, nutrient retention, and microbial habitat stability (Jiang et al., 2025a; Kukowska and Szewczuk-Karpisz, 2025). These changes can affect microbial growth and P transformation (Pang et al., 2024). Microbial processes are crucial for soil P cycling, and *phoD* gene abundance is widely recognized as a sensitive biomarker of organic P mineralization in soil; therefore, it can be used alongside phosphatase activity to evaluate biological P regulation (Wang et al., 2021). An integrated analysis of soil properties, P fractions, microbial traits, and plant P nutrition is therefore needed to understand how nano-zeolite-coupled biochar-based P fertilizer works in acidic Moso bamboo soils.

To clarify these unresolved issues, a two-year field trial was established in a subtropical Moso bamboo forest to determine whether nano-zeolite coupling strengthened the effects of biochar-based phosphorus fertilizer on soil P availability, microbial P cycling, and leaf P concentration relative to conventional phosphorus fertilizer (CP) and unmodified biochar-based phosphorus fertilizer (BP). Specifically, the present research aimed to: (1) determine the temporal dynamics of soil P forms under different P fertilizer treatments; (2) evaluate impacts of BP and NBP on soil pH, microbial biomass P, phosphatase activity, and *phoD* gene abundance; and (3) determine whether alterations in soil P availability and microbially mediated P cycling were linked to changes in leaf P status of Moso bamboo. We hypothesized that biochar-based P fertilizers would enhance soil P availability by alleviating soil acidity, thereby reducing the accumulation of poorly available inorganic P fractions and maintaining more P in labile and moderately labile pools. Meanwhile, these fertilizers were expected to stimulate microbial P turnover by increasing *phoD* gene abundance and alkaline phosphatase activity. We further hypothesized that nano-zeolite coupling would strengthen these effects by improving nutrient retention through its microporous structure and ion-exchange capacity, while providing a more stable microbial habitat that promotes organic P mineralization. These combined physicochemical and biological effects were expected to result in greater and more sustained improvements in soil P availability and leaf P concentration.

## Materials and methods

### Site description

The study was carried out in a Moso bamboo plantation. It was located in Hangzhou, Zhejiang Province, China (30°13′N, 119°47′E). The region has a subtropical monsoon climate, with warm temperatures and abundant rainfall. During the study, the annual precipitation was about 1459 mm, and the annual temperature was 19.03 °C. The current Moso bamboo stand was established in 2009 on a site that was previously covered by natural broad-leaved forest. At the time of the study, the stand density was approximately 2,985 culms ha^−1^, with a mean diameter at breast height of 9.7 cm.

Before this experiment, the bamboo forest received fertilizer every year. It received urea, calcium superphosphate, and potassium chloride. These fertilizers supplied 200 kg N ha^−1^, 57 kg P ha^−1^, and 66 kg K ha^−1^. Farmers cleared the small ground plants every August. We stopped adding fertilizer in July 2021 to reduce the carry-over effect of earlier fertilizer inputs. We tested the topsoil (0–20 cm) before the treatments. The soil layer had a pH of 4.84, soil organic carbon of 18.85 g kg^−1^, total nitrogen of 1.82 g kg^−1^, available phosphorus of 7.61 mg kg^−1^, and available potassium of 89.4 mg kg^−1^.

### Experimental design

The field trial was carried out in a Moso bamboo forest, and the test ran from August 2023 to August 2025. The study included four treatments: an unfertilized control, conventional P fertilizer (CP), biochar-based P fertilizer (BP), and nano-zeolite-coupled biochar-based P fertilizer (NBP). The CP, BP, and NBP treatments were applied at an identical P rate of 31.5 kg P ha^−1^.

A randomized complete block design was used, with three replicates per treatment and 12 plots in total. The blocking factor was a sloping gradient, which was used to minimize the potential influence of environmental heterogeneity among plots. Each plot measured 10 m × 10 m, corresponding to an area of 100 m^2^. Soil and leaf samples were collected at months 1, 12, and 24 after fertilizer application.

### Sample collection and preparation

Surface soil samples (0–20 cm) were obtained at 1, 12, and 24 months following fertilizer application. In each plot, five soil cores were collected using a five-point sampling method and composited into one representative sample. Collected soils were sealed, stored in a cooled container, and transported to the laboratory. Fresh subsamples were passed through a 2 mm sieve for analyses of microbial biomass phosphorus (MBP), *phoD* gene abundance, and acid and alkaline phosphatase activities. The remaining soil was air-dried. A portion was sieved through a 2 mm mesh for the measurement of soil pH, while another portion was further ground and sieved through a 0.149 mm mesh for the determination of soil P fractions.

For leaf sampling, three representative Moso bamboo culms were selected in each plot. The selected culms were healthy and had diameters close to the mean diameter at breast height of the plot. Current-year, fully expanded, healthy mature leaves were collected from the upper-middle part of the outer canopy of each selected culm. Approximately 20–30 leaves were sampled from each selected culm. Leaves from the three culms in each plot were pooled, transported to the laboratory, rinsed, oven-dried, ground, and sieved for total leaf P analysis.

### Measurement of soil phosphorus fractions

Soil P fractionation was performed using a modified Hedley sequential extraction method. Briefly, 1.0 g of air-dried soil (<0.149 mm) was extracted with 30 mL of ultrapure water in a centrifuge tube by shaking for 16 h, followed by centrifugation for 10 min. The supernatant was collected and subsequently analyzed to measure the concentration of water-extractable inorganic P (H_2_O-P_i_).

After water extraction, the remaining soil was sequentially extracted with 30 mL of 0.5 mol L^–1^ NaHCO_3_ (pH 8.5), 30 mL of 0.1 mol L^–1^ NaOH, and 30 mL of 1.0 mol L^–1^ HCl. Each extraction was carried out by shaking for 16 h, followed by centrifugation for 10 min. The supernatant was then collected. Inorganic P in the NaHCO_3_ and NaOH extracts was determined directly as NaHCO_3_-P_i_ and NaOH-P_i_. Total P in these extracts was measured after digesting 10 mL aliquots with ammonium persulfate [(NH_4_)_2_S_2_O_8_] at 120–124 °C for 30 min. Organic P was calculated as the difference between total and inorganic P for the corresponding extracts. The HCl extract was used to determine HCl-P_i_. After sequential extraction, the remaining residue was transferred to a digestion tube and digested with H_2_SO_4_–HClO_4_ at 360 °C to determine residual P. Phosphorus concentrations in all extracts and digests were measured colorimetrically.

### Measurement of soil pH and microbial biomass phosphorus

Soil pH was measured in a 1:2.5 soil–water suspension using a pH meter. MBP was analyzed using the chloroform fumigation–extraction method. Briefly, paired 2.0 g fresh soil subsamples were prepared for each sample; one was fumigated with ethanol-free chloroform vapor in a vacuum desiccator for 24 h in the dark, and the other was used as an unfumigated control. After removing residual chloroform, both subsamples were extracted with NaHCO_3_ solution. The extracts were shaken, centrifuged, and filtered through phosphorus-free filter paper. Phosphorus concentration in the extracts was measured colorimetrically. MBP was estimated as the increase in NaHCO_3_-extractable P released by chloroform fumigation relative to the unfumigated control.

### Measurement of soil phosphatase activity

Acid and alkaline phosphatase activities were determined according to a modified Tabatabai and Bremner (1969) method. Fresh soil (1.00 g) was placed in a 50 mL Erlenmeyer flask and mixed with 0.2 mL toluene and 4 mL modified universal buffer at pH 6.5 for acid phosphatase or pH 11.0 for alkaline phosphatase. Then, 1 mL of 0.05 mol L^−1^ *p*-nitrophenyl phosphate solution made in the same buffer was added into the flask. After brief mixing, the flasks were capped and incubated at 37 °C for 1 h. The reaction was stopped by adding 1 mL of 0.5 mol L^−1^ CaCl_2_ and 4 mL of 0.5 mol L^−1^ NaOH. The soil suspension was subsequently shaken, filtered, and measured spectrophotometrically at 405 nm.

### Soil *phoD* gene abundance

Soil genomic DNA was extracted from 0.20 g fresh soil with a soil DNA extraction kit (M5635-02; Omega Bio-Tek, Norcross, GA, USA) and stored at −20 °C until analysis. The *phoD* gene abundance was quantified by quantitative PCR using ALPS-F730 (5′-CAGTGGGACGACCACGAGGT-3′) and ALPS-1101 (5′-GAGGCCGATCGGCATGTCG-3′) as primers. The PCR program included an initial 5 min denaturation at 98 °C, five cycles of 98 °C for 30 s, 58 °C for 30 s, and 72 °C for 45 s, followed by 28 cycles of 98 °C for 30 s, 55 °C for 30 s, and 72 °C for 45 s, and a final extension at 72 °C for 5 min. Product specificity was checked by melting curve analysis and agarose gel electrophoresis. Gene copy numbers were calculated using plasmid-based standard curves and reported as copies g^−1^ dry soil.

### Leaf phosphorus concentration

Leaf P concentration was determined after acid digestion. In brief, dried leaf powder (0.20 g) was digested with nitric acid–persulfate solution until clear. The cooled digest was filtered, diluted to volume with deionized water, and analyzed for P using the ammonium molybdate colorimetric method.

### Statistical analysis

Data were entered in Microsoft Excel 2016 and analyzed with IBM SPSS Statistics 26. Before ANOVA, all data were tested for normality and homogeneity of variance. Because the data satisfied these assumptions, no mathematical transformation was applied. Treatment effects were tested separately for each sampling date using one-way ANOVA. The tested variables included soil P fractions, soil physicochemical properties, phosphatase activities, *phoD* gene abundance, and leaf P concentration. When ANOVA showed a significant treatment effect, treatment means were separated with the LSD test at *P* < 0.05. Pearson correlation was used to test the links between phosphatase activities and soil P fractions. Figures were drawn with Origin Pro 2021.

## Results

### Soil phosphorus fractions

**Figure 1:**
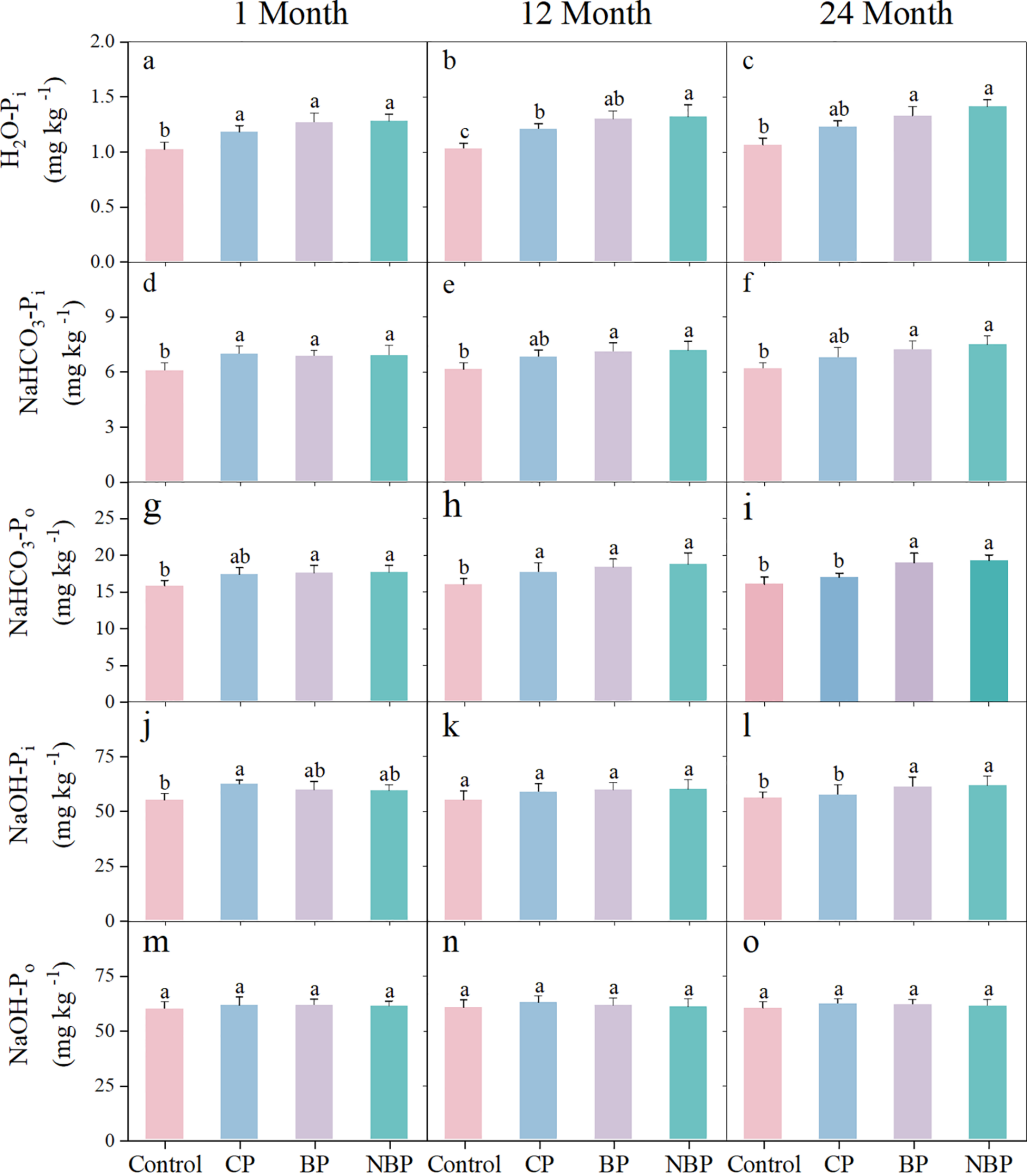
Impacts of different fertilizer treatments on soil **(a–c)** H_2_O-P_i_, **(d–f)** NaHCO_3_-P_i_, **(g–i)** NaHCO_3_-P_o_, **(j–l)** NaOH-P_i_, and **(m–o)** NaOH-P_o_ fractions. Treatments include: Control, no fertilizer; CP, phosphorus fertilizer; BP, biochar-based phosphorus fertilizer; NBP, nano-zeolite coupled biochar-based phosphorus fertilizer. Values are presented as means ± standard deviations. Lowercase letters denote significant differences among treatments at *P* < 0.05.

**Figure 2:**
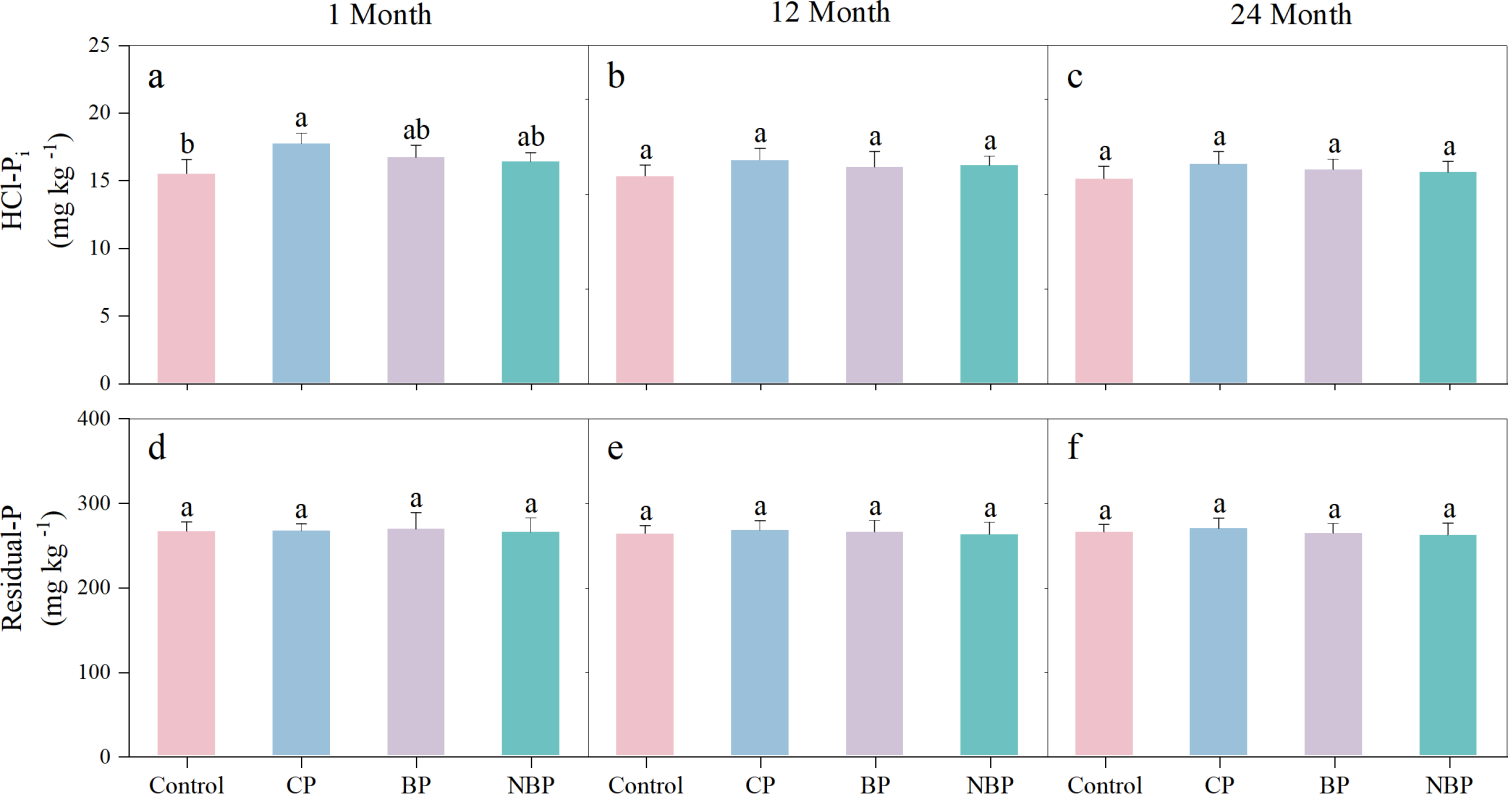
Impacts of different fertilizer treatments on soil **(a–c)** HCl-P_i_, **(d–f)** Residual-P fractions. Treatments include: Control, no fertilizer; CP, phosphorus fertilizer; BP, biochar-based phosphorus fertilizer; NBP, nano-zeolite coupled biochar-based phosphorus fertilizer. Values are presented as means ± standard deviations. Lowercase letters denote significant differences among treatments at *P* < 0.05.

Soil P forms varied among treatments and sampling times, with the strongest responses observed in the labile P pools (**Figures 1**, **2**). Across most sampling dates, all fertilization treatments increased H_2_O-P_i_ and NaHCO_3_-P_i_ concentrations compared with the control (**Figures 1a–f**). By month 24, NBP produced the highest H_2_O-P_i_ and NaHCO_3_-P_i_ concentrations, although these values did not differ significantly from those under BP.

Fractions of NaHCO_3_-P_o_ and NaOH-P_i_ were also affected by fertilization (**Figures 1g–l**). The BP and NBP treatments generally outperformed the CP treatment over time. At month 24, both BP and NBP significantly increased NaHCO_3_-P_o_ and NaOH-P_i_ concentrations relative to the control and CP treatments (**Figures 1g–l**). In contrast, fertilization had limited effects on more stable P pools, as NaOH-P_o_ and residual-P showed no significant differences among treatments (**Figures 1m–o**, **2d–f**). Notably, CP consistently produced the highest HCl-P_i_ concentration, whereas BP and NBP limited the accumulation of this poorly available fraction, with no significant increases relative to the control (**Figures 2a–c**).

### Soil pH and microbial biomass phosphorus

**Figure 3:**
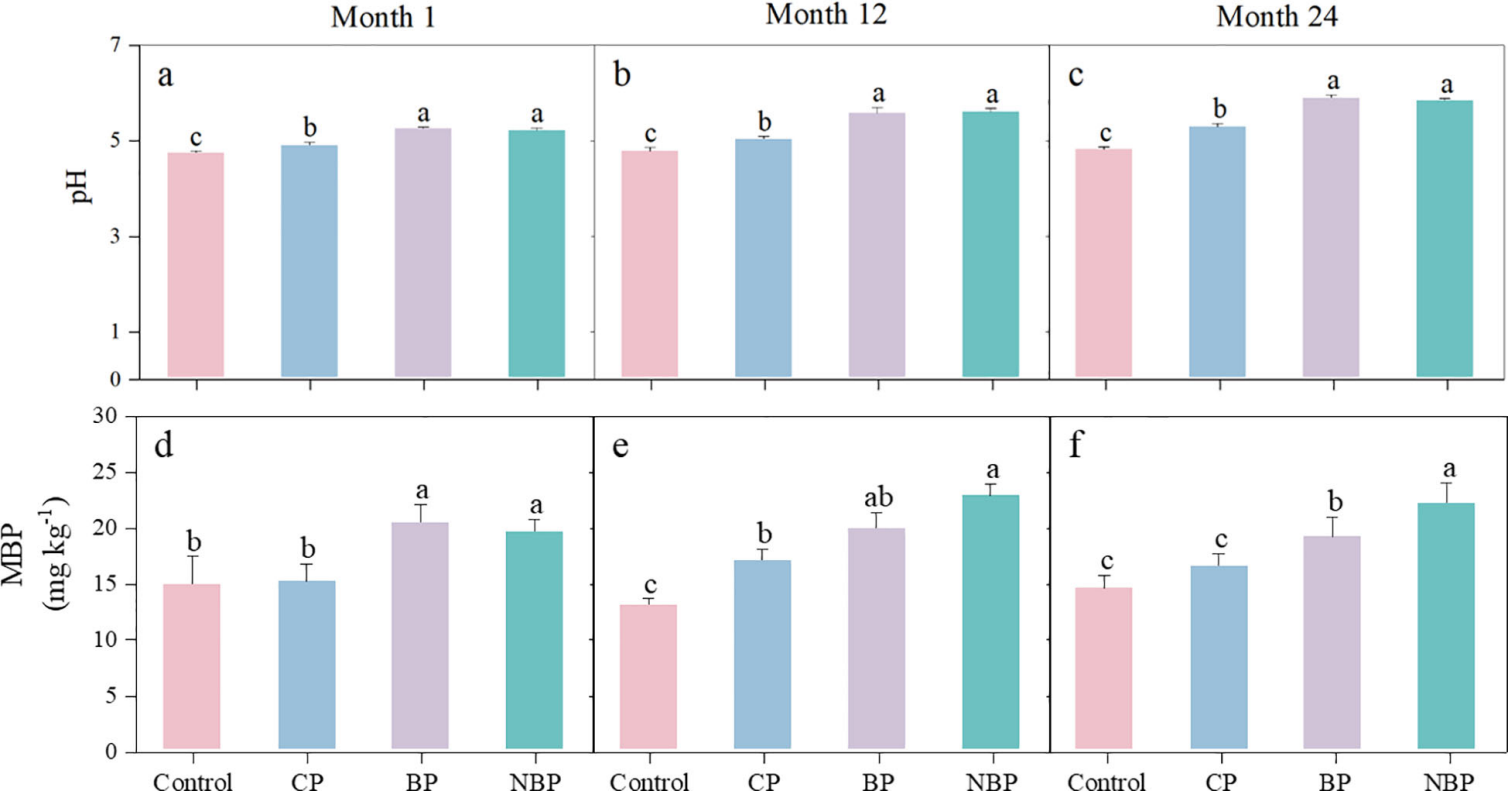
Impacts of different fertilizer treatments on soil **(a–c)** pH and **(d–f)** microbial biomass phosphorus (MBP). Treatments include: Control, no fertilizer; CP, phosphorus fertilizer; BP, biochar-based phosphorus fertilizer; NBP, nano-zeolite coupled biochar-based phosphorus fertilizer. Values are presented as means ± standard deviations. Lowercase letters denote significant differences among treatments at *P* < 0.05.

Fertilization significantly affected soil pH and MBP concentration (**Figure 3**). Soil pH was higher under all fertilized treatments relative to the control, with higher values observed under BP and NBP than under CP (**Figures 3a–c**). Soil pH was significantly higher under BP and NBP compared with the control and CP treatments at month 1, and this effect persisted until month 24. At the final sampling, BP and NBP maintained significantly higher pH values than the control and CP treatments, but did not differ significantly from each other (**Figure 3c**).

The MBP concentration showed a clear response to biochar-based phosphorus fertilizers. At month 1, the BP and NBP treatments significantly increased MBP concentration, whereas the CP treatment did not differ significantly from the control (**Figure 3d**). At month 12, the MBP concentration was highest under NBP and was considerably greater than that under control and CP, while BP also maintained a relatively high MBP level (**Figure 3e**). By month 24, NBP still resulted in the highest MBP concentration and was significantly higher than BP, CP, and the control (**Figure 3f**), indicating a persistent positive effect of NBP on the microbial P pool.

### Soil phosphatase activity

**Figure 4:**
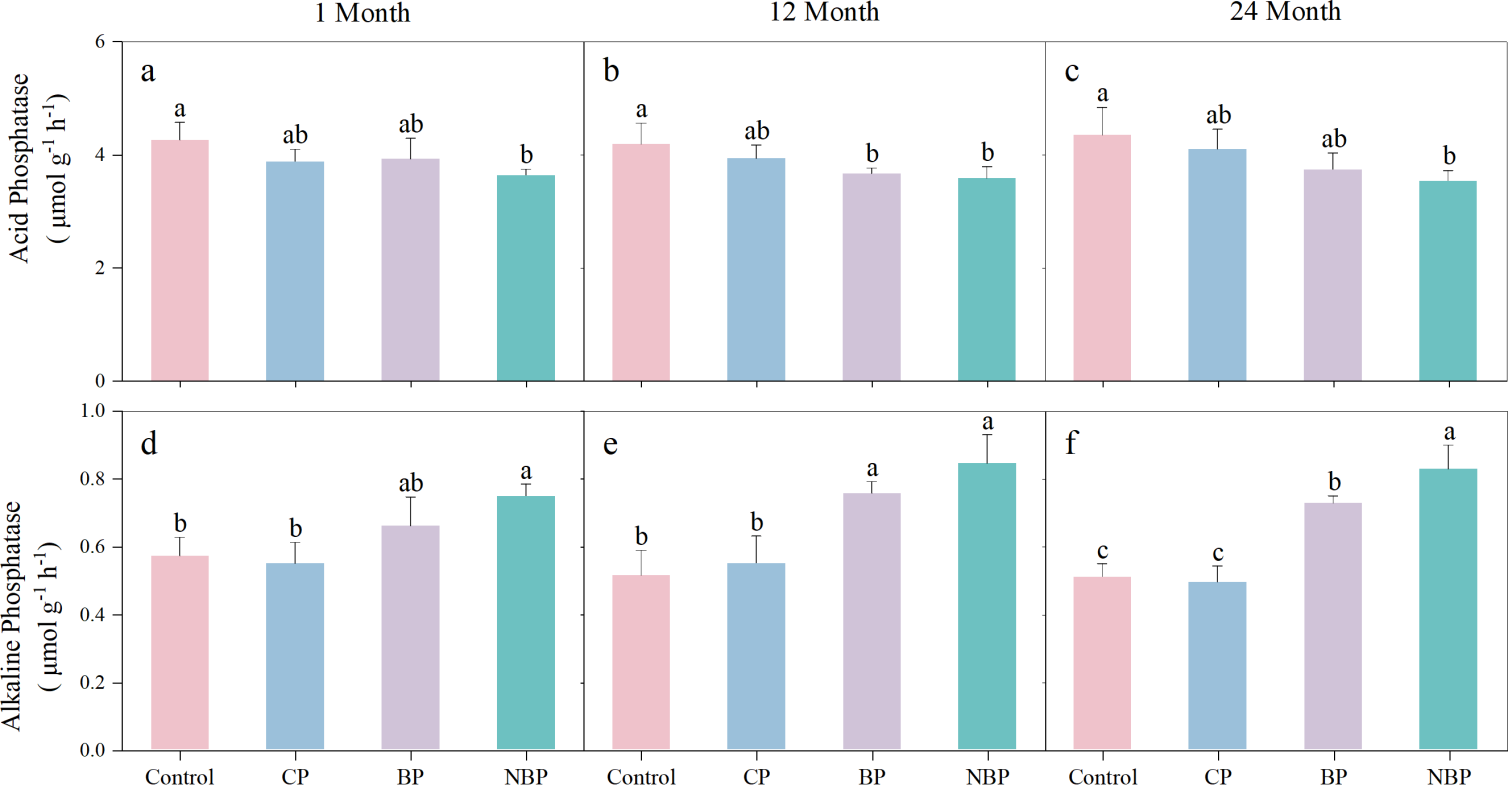
Impacts of different fertilizer treatments on soil **(a–c)** acid phosphatase (ACP) and **(d–f)** alkaline phosphatase (ALP) activities. Treatments include: Control, no fertilizer; CP, phosphorus fertilizer; BP, biochar-based phosphorus fertilizer; NBP, nano-zeolite coupled biochar-based phosphorus fertilizer. Values are presented as means ± standard deviations. Lowercase letters denote significant differences among treatments at *P* < 0.05.

Acid and alkaline phosphatase activities responded differently to fertilizer application (**Figure 4**). Fertilization generally suppressed acid phosphatase activity, with the most pronounced decline occurring under the NBP treatment (**Figures 4a–c**). At month 1, acid phosphatase activity under NBP was significantly lower than that under the control (**Figure 4a**). At months 12 and 24, NBP continued to maintain relatively low acid phosphatase activity (**Figures 4b, c**). In comparison, CP and BP had weaker effects on acid phosphatase activity.

Alkaline phosphatase activity showed the opposite response, being significantly enhanced under BP and NBP but not under CP relative to the control (**Figures 4d–f**). At the first sampling, the NBP treatment resulted in significantly greater alkaline phosphatase activity than the control and CP treatments (**Figure 4d**). At month 12, both BP and NBP treatments exhibited significantly higher alkaline phosphatase activity, with NBP treatment maintaining a relatively high level (**Figure 4e**). By month 24, alkaline phosphatase activity was highest under NBP treatment and was significantly higher than that under BP treatment, while BP treatment was also significantly higher than the control and CP treatments (**Figure 4f**). These results indicate that biochar-based phosphorus fertilizers, particularly NBP, enhanced soil alkaline phosphatase activity over time.

### Soil *phoD* abundance

**Figure 5:**
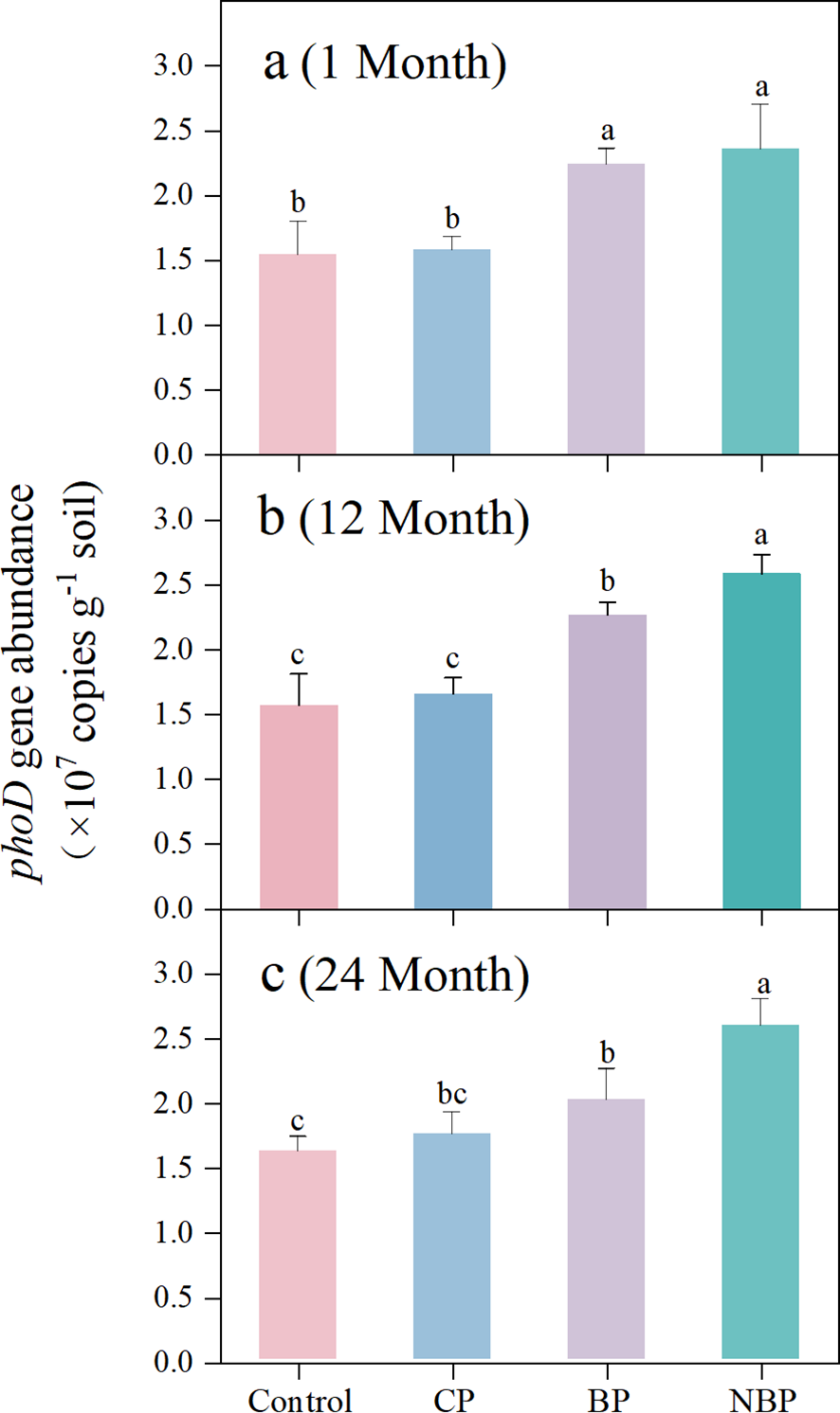
Impacts of different fertilizer treatments on soil *phoD* gene abundance at **(a)** month 1, **(b)** month 12, and **(c)** month 24. Treatments include: Control, no fertilizer; CP, phosphorus fertilizer; BP, biochar-based phosphorus fertilizer; NBP, nano-zeolite coupled biochar-based phosphorus fertilizer. Values are presented as means ± standard deviations. Lowercase letters denote significant differences among treatments at *P* < 0.05.

Soil *phoD* abundance was significantly affected by fertilizer type (**Figure 5**). The CP treatment had a relatively weak effect on *phoD* abundance and did not differ significantly from the control at most sampling times (**Figure 5**). In contrast, the *phoD* abundance was significantly higher under the BP and NBP treatments, with the strongest effect observed under NBP treatment (**Figure 5**).

At month 1, BP and NBP treatments enhanced *phoD* abundance relative to the control and CP treatments, with no difference between the two biochar-based fertilizers (**Figure 5a**). At month 12, the *phoD* abundance increased further under NBP treatment and was higher than in all other treatments (**Figure 5b**). This pattern remained at month 24. The NBP treatment had the highest *phoD* abundance, while BP treatment was higher than the control but lower than NBP (**Figure 5c**). These results indicate a sustained increase in *phoD*-harboring microorganisms under the NBP treatment.

### Leaf phosphorus concentration

**Figure 6:**
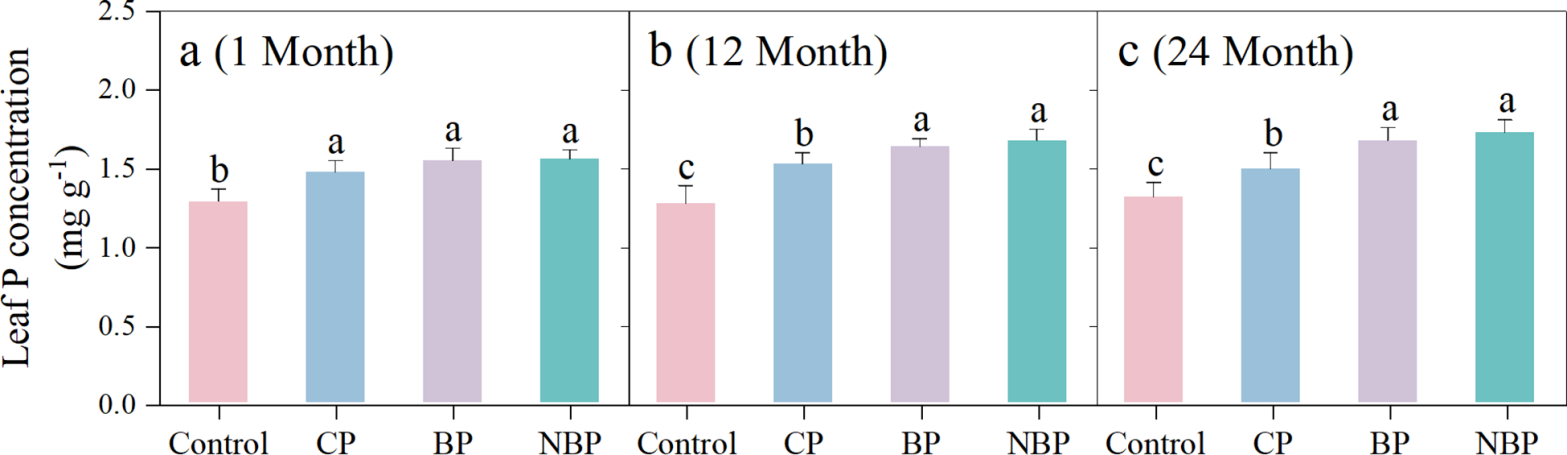
Impacts of different fertilizer treatments on leaf phosphorus concentration of Moso bamboo at **(a)** month 1, **(b)** month 12, and **(c)** month 24. Treatments include: Control, no fertilizer; CP, phosphorus fertilizer; BP, biochar-based phosphorus fertilizer; NBP, nano-zeolite coupled biochar-based phosphorus fertilizer. Values are presented as means ± standard deviations. Lowercase letters denote significant differences among treatments at *P* < 0.05.

Fertilization enhanced the leaf P concentration in the Moso bamboo plant, but the response changed with sampling time (**Figure 6**). At month 1, all fertilization treatments showed higher leaf P concentration than the control. No significant difference was found among the fertilization treatments (**Figure 6a**).

Differences among treatments became more pronounced at the later sampling stages. By months 12 and 24, BP and NBP consistently outperformed CP, with NBP maintaining the highest leaf P concentration at both time points (**Figures 6b, c**). These results indicate that biochar-based P fertilizers, particularly NBP, were more effective than conventional P fertilizer in sustaining long-term leaf P nutrition.

### Relationships between phosphatase activities and soil phosphorus fractions

**Figure 7:**
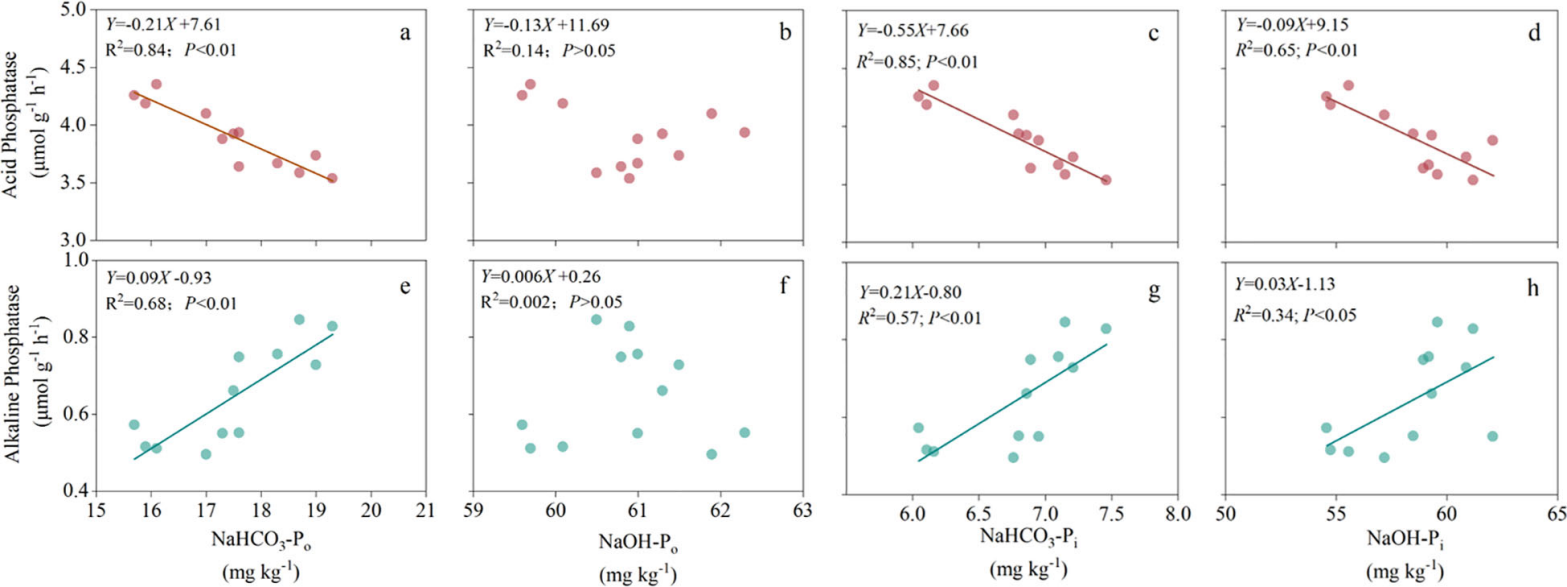
Correlation between phosphatase activities and different soil phosphorus fractions. **(a–d)** acid phosphatase (ACP) and soil phosphorus fractions, and **(e–h)** alkaline phosphatase (ALP) and soil phosphorus fractions.

Phosphatase activities showed distinct relationships with different soil P fractions (**Figure 7**). The acid phosphatase activity was significantly negatively associated with NaHCO_3_-P_o_, NaHCO_3_-P_i_, and NaOH-P_i_, with the strongest negative relationship observed between acid phosphatase activity and NaHCO_3_-P_i_ (R^2^ = 0.85, *P* < 0.01) (**Figures 7a, c, d**). The acid phosphatase activity was not associated with NaOH-P_o_ (**Figure 7b**).

Alkaline phosphatase activity was positively related to NaHCO_3_-P_o_, NaHCO_3_-P_i_, and NaOH-P_i_, with the strongest association observed for NaHCO_3_-P_o_ (R^2^ = 0.68, *P* < 0.01), followed by NaHCO_3_-P_i_ (R^2^ = 0.57, *P* < 0.01) and NaOH-P_i_ (R^2^ = 0.34, *P* < 0.05) (**Figures 7e, g, h**). No significant relationship was detected between alkaline phosphatase activity and NaOH-P_o_ (**Figure 7f**). These results indicate distinct associations of acid and alkaline phosphatases with soil P fractions, and highlight the closer positive linkage between alkaline phosphatase and labile or moderately labile P pools.

## Discussion

### Biochar-based phosphorus fertilizers improved soil pH and maintained labile phosphorus pools

Our results demonstrated that biochar-based fertilizers, particularly the NBP treatment, effectively improved soil P availability by raising soil pH, enhancing labile and moderately labile P fractions, and restricting the accumulation of less accessible inorganic P. Soil pH controls phosphorus availability in acidic soils by regulating P fixation, desorption, and transformation (Wang et al., 2023a). In this study, soil pH increased across all fertilized treatments, with the biochar-based fertilizers producing larger increases than conventional phosphorus fertilizer (**Figures 3a–c**). This response is consistent with the alkaline nature of biochar and its capacity to neutralize soil acidity through ash alkalinity, surface functional groups, and proton-consuming reactions (Glaser and Lehr, 2019; Bolan et al., 2023). In acidic soils, soluble phosphate ions are readily adsorbed or precipitated by Fe and Al oxides through ligand exchange, thereby forming less available inorganic P pools (Chen et al., 2022; He et al., 2025). The consistently higher HCl-P_i_ concentration under CP treatment in our study suggests that conventional phosphorus fertilizer promoted the transformation of added P into relatively stable inorganic fractions (Cordeiro and Rosolem, 2026). In contrast, BP and NBP maintained lower HCl-P_i_ concentration than CP, implying that biochar-based fertilizers suppressed the transformation of fertilizer-derived P into less accessible soil P fractions (Alotaibi et al., 2021).

The increase in soil pH under BP and NBP treatments likely weakened the binding strength between phosphate and Fe/Al oxides, thereby favoring the maintenance of more available P fractions (Ma et al., 2025). Biochar can alleviate Al toxicity and reduce phosphate fixation by increasing pH, complexing Al^3+^, and modifying the surface chemistry of soil minerals (Qian et al., 2013; Glaser and Lehr, 2019; Ghodszad et al., 2021). During the early and middle stages of the experiment, these processes may explain the higher H_2_O-P_i_ and NaHCO_3_-P_i_ concentrations observed under BP and NBP compared with CP. The stronger effect of NBP, particularly at month 24, further suggests that nano-zeolite coupling enhanced the ability of biochar-based fertilizer to maintain labile inorganic P pools over time. Owing to its large surface area, abundant microporous channels, and strong ion-exchange properties, nano-zeolite can enhance nutrient retention and slow nutrient depletion from the soil–fertilizer system (Nakhli et al., 2017; Jarosz et al., 2022; Dong et al., 2025; Jiang et al., 2026a). When combined with biochar, these properties may create a more stable physicochemical environment that buffers soil acidity and regulates P adsorption–desorption processes (Chen et al., 2024a).

In addition to inorganic labile P, NBP also increased NaHCO_3_-P_o_ and NaOH-P_i_ concentrations, especially during the later stages of the experiment. The NaHCO_3_-P_o_ is generally regarded as a relatively labile organic P fraction, while NaOH-P_i_ represents Fe/Al-associated P with intermediate availability. The NBP treatment increased these phosphorus fractions. This result shows that nano-zeolite-coupled biochar-based fertilizer did not simply increase immediately soluble P. It also redistributed P into pools that keep it available over time (Li et al., 2023). This redistribution is closely associated with the unique physicochemical properties of the nano-zeolite–biochar matrix. The high cation-exchange capacity and abundant microporous channels of nano-zeolite can facilitate nutrient retention and regulate interactions between phosphate and metal ions, thereby favoring the redistribution of added P toward labile and moderately labile fractions rather than its fixation into more stable mineral-associated pools (Hasbullah et al., 2020). Meanwhile, the relatively stable microenvironment provided by the nano-zeolite–biochar matrix may promote microbial proliferation and phosphatase secretion, thereby accelerating soil P transformation and turnover (Galamini et al., 2025; Wu et al., 2026). This steady supply was important for Moso bamboo forests, where P is needed throughout the growing seasons. The fertilizer treatment had no significant effect on residual-P. This indicates that the fertilizer treatment affected the labile and moderately labile P pools, rather than the highly stable residual-P fraction.

### NBP enhanced microbial phosphorus retention and biological phosphorus turnover

Our results further revealed that the NBP treatment profoundly enhanced microbial P cycling, evidenced by increased MBP, elevated alkaline phosphatase activity, and higher *phoD* gene abundance. The BP and NBP treatments enhanced MBP concentration, and the NBP treatment showed the strongest effect. This indicates that biochar-based P fertilizers help microbes catch and recycle phosphorus (Spohn and Widdig, 2017; Fei et al., 2025). Microbial retention may transiently lower soluble inorganic P. At the same time, it can prevent P from being transformed into poorly available mineral pools or being lost from soil. When microbes die, lyse, and decompose, they release the P they hold. This process helps maintain a steady supply of available P in the soil over time (Richardson and Simpson, 2011; Spohn and Widdig, 2017; Fei et al., 2025).

The NBP fertilizer improved the soil condition. It also created a more stable living space for microbes. These changes help explain the higher microbial phosphorus. Biochar is highly porous, providing a habitat for microbial growth. Its surface functional groups can hold nutrients, and also help protect extracellular enzymes (Lehmann et al., 2011; Gul and Whalen, 2016). Nano-zeolite makes these benefits even stronger. Its microporous structure and high cation-exchange capacity work together. They improve nutrient retention and help stabilize the microbial microenvironment (Elzobair et al., 2016; Nakhli et al., 2017; Jarosz et al., 2022). Working together, biochar and nano-zeolite may have helped microorganisms access P and store more P in microbial biomass (Hou et al., 2022).

The NBP treatment had different effects on acid and alkaline phosphatase activities. Acid phosphatase activity declined, but alkaline phosphatase activity increased. This change may lead to a shift in microbial phosphorus turnover. The acid phosphatase activity decreased, which may be related to increase in soil pH and the higher availability of inorganic P. Acid phosphatase is more active in acidic environments. When available phosphorus increases, microbes may reduce its secretion (Foster et al., 2016; Cui et al., 2022; Jiang et al., 2026b). By contrast, the higher alkaline phosphatase activity under NBP treatment was consistent with increased soil pH and MBP. Alkaline phosphatase breaks down organic matter to release phosphorus. It actively helps microbes recycle nutrients when the soil pH is suitable (Li et al., 2021).

The NBP treatment increased the abundance of the *phoD* gene. This also reflects an enhanced microbial potential for phosphorus mineralization. The *phoD* gene encodes alkaline phosphatase. It is often used as a marker for microbial organic P mineralization (Ragot et al., 2015; Wang et al., 2021). Under NBP treatment, *phoD* abundance and alkaline phosphatase activity increased at the same time. This indicates that nano-zeolite coupling affected both the functional-gene level and the enzyme-activity level. Similar responses have been reported in subtropical forest soils with added biochar (Xiao et al., 2026). Biochar increased microorganisms carrying the *phoD* gene. It also enhanced biological phosphorus turnover (Tian et al., 2021; Wang et al., 2023b; Jiang et al., 2025b). In our study, MBP, *phoD* abundance, and alkaline phosphatase activity all increased under NBP treatment. This suggests that soil P supply does not rely only on direct fertilizer input. Microbial retention and mineralization also play an important role (Chen et al., 2024b).

### Relationships between phosphatase activities and active phosphorus pools

Our data showed that acid and alkaline phosphatases connect to soil P fractions in different ways. Acid phosphatase activity declined as NaHCO_3_-P_i_, NaHCO_3_-P_o_, and NaOH-P_i_ increased. Alkaline phosphatase activity showed the opposite trend. Its activity grew along with these phosphorus fractions. This opposite trend shows a clear result. The two enzymes act in completely different ways. They react differently when the soil’s phosphorus and pH change.

The acid phosphatase activity was negatively related to labile and moderately labile P fractions. This result means that microbes need less acid phosphatase when the soil already has enough available P. In P-limited acidic soils, microbes and plant roots often produce more acid phosphatase to release P from organic compounds (Chen et al., 2026). Under biochar-based fertilizer treatments, available P increased and soil pH also rose. These changes may decrease the level of acid phosphatase in the soil (Foster et al., 2016; Cui et al., 2022). This situation matches our findings. Under the NBP treatment, the acid enzyme level was lower. However, the available P and soil pH were higher.

In contrast, alkaline phosphatase activity was most closely related to NaHCO_3_-P_o_. Its relationships with NaOH-P_i_ and NaHCO_3_-P_i_ were also positive, but weaker. This indicates that alkaline phosphatase was linked to the turnover of labile and moderately labile P pools under biochar-based fertilizer treatments. The positive link with NaHCO_3_-P_o_ also points to organic P mineralization as a source of available P (Li et al., 2021). Under NBP, alkaline phosphatase activity increased strongly. This enzyme pathway may therefore help explain why NBP maintained soil P availability during the 24-month experiment.

### Effect of soil phosphorus availability on leaf phosphorus nutrition in the Moso bamboo plant

Our study indicates that conventional phosphorus fertilizers provide only short-term effects, whereas biochar-based fertilizers, especially NBP, can supply phosphorus to Moso bamboo continuously, as reflected in leaf P concentrations (Tougaard et al., 2023; Shen et al., 2024). In this trial, all fertilizer treatments increased leaf P concentration at month 1. This indicates that Moso bamboo responded quickly to P addition. The effect of the CP treatment became weaker at later sampling dates. In contrast, the BP and NBP treatments kept leaf P concentration increasing. This suggests that the CP treatment mainly supplied P in the short term. The BP and NBP treatments were more effective in maintaining P supply in acidic Moso bamboo soils.

The weaker effect of CP on leaf P concentration was consistent with the higher HCl-P_i_ level under the CP treatment. This suggests that part of the P was transformed into poorly available inorganic pools. As a result, less P was available for long-term plant uptake. But the BP and NBP treatments showed a different pattern. They maintained higher H_2_O-Pi, NaHCO_3_-P_i_ and NaOH-P_i_ pools. They also enhanced MBP and biological P turnover. These changes gave the bamboo roots a steady supply of P. This benefit showed up in the leaf P levels (Richardson and Simpson, 2011; Spohn and Widdig, 2017).

The NBP treatment supported leaf P concentration better than BP treatment. This may be due to the joint effect of nano-zeolite and biochar. Biochar can adsorb soluble P and reduce P leaching. It can also release part of the retained P later through desorption and microbial processes (Ghodszad et al., 2021; Luo et al., 2023). Nano-zeolite can enhance the characteristics of nutrient retention, water holding capacity, and ion exchange, thereby strengthening the slow-release characteristics of the fertilizer system (Khan et al., 2021; Jarosz et al., 2022; Dong et al., 2025). Additionally, the NBP treatment may also have created a more stable microbial habitat. This could support *phoD*-harboring microorganisms and alkaline phosphatase activity, and promote biological P mineralization (Elzobair et al., 2016). These changes help explain the higher leaf P concentration under NBP treatment at the later sampling stages.

### Phosphorus management in acidic Moso bamboo soils

Our results show that NBP improved P availability in acidic Moso bamboo soils through both soil chemical changes and microbial processes. Conventional P fertilization mainly increased soil P input, but a substantial portion of this P appeared to be transformed into HCl-P_i_, limiting its long-term availability. In contrast, the NBP treatment increased soil pH, maintained labile and moderately labile P fractions, enhanced MBP accumulation, stimulated *phoD* abundance and alkaline phosphatase activity, and ultimately improved leaf P concentration.

These findings suggest that the advantage of NBP does not rely solely on direct nutrient release from the fertilizer. Instead, NBP appears to reorganize soil P cycling by coupling chemical P retention with microbial P turnover. This mechanism is particularly valuable in acidic subtropical soils, where rapid immobilization of phosphate by Fe and Al oxides frequently constrains the effective use of applied P fertilizers (Cordeiro and Rosolem, 2026). By reducing the transformation of applied P into less available pools and promoting biologically mediated P recycling, NBP may better synchronize soil P supply with the long-term nutrient demand of perennial Moso bamboo forests (Gul and Whalen, 2016; Qin et al., 2025).

For practical nutrient management, nano-zeolite-coupled biochar-based phosphorus fertilizer may offer an effective approach to enhance P-use efficiency and lessen reliance on conventional P inputs in acidic bamboo forest ecosystems (Zhu et al., 2017; Tian et al., 2021; Liu et al., 2026). However, further work is needed to evaluate its long-term field performance, optimal application rates, effects on P leaching losses, and interactions with microbial community composition. Longer field trials, together with microbial community and functional analyses, are necessary to test how NBP regulates P cycling across different soils and climates.

## Conclusions

This field study revealed that the NBP treatment improved P availability in acidic Moso bamboo soils more effectively than conventional P fertilizer and biochar-based P fertilizer. The NBP treatment raised soil pH and maintained more P in labile and moderately labile forms, including H_2_O-P_i_, NaHCO_3_-P_i_, NaHCO_3_-P_o_, and NaOH-P_i_. It also reduced HCl-P_i_ accumulation compared with conventional P fertilizer. This suggests that less applied P was transformed into poorly available inorganic pools. The NBP treatment also increased microbial biomass P, *phoD* gene abundance, and alkaline phosphatase activity. These changes point to stronger microbial P retention and organic P mineralization. Higher soil P availability was also reflected in higher leaf P concentration, especially at the later sampling stages. In summary, NBP improved soil P supply by regulating soil pH, retaining more available P, and strengthening microbial P turnover. These findings suggest that nano-zeolite-coupled biochar-based phosphorus fertilizer has potential for enhancing soil P availability and supporting sustainable nutrient management in acidic Moso bamboo forest ecosystems.

## Data Availability

The original contributions presented in the study are included in the article/supplementary material. Further inquiries can be directed to the corresponding author.
